# Aquaporin 1 and 5 expression decreases during human intervertebral disc degeneration: novel HIF-1-mediated regulation of aquaporins in NP cells

**DOI:** 10.18632/oncotarget.3631

**Published:** 2015-03-20

**Authors:** Zariel I. Johnson, Shilpa S. Gogate, Rebecca Day, Abbie Binch, Dessislava Z. Markova, Neil Chiverton, Ashley Cole, Matt Conner, Irving M. Shapiro, Christine L. Le Maitre, Makarand V. Risbud

**Affiliations:** ^1^ Department of Orthopaedic Surgery and Graduate Program in Cell and Developmental Biology, Thomas Jefferson University, Philadelphia, PA, USA; ^2^ Biomedical Research Centre, Sheffield Hallam University, Sheffield, UK; ^3^ Sheffield Teaching Hospitals NHS Foundation Trust, Sheffield, UK

**Keywords:** intervertebral disc, nucleus pulposus, aquaporin 1, aquaporin 5, HIF-1

## Abstract

Objectives of this study were to investigate whether AQP1 and AQP5 expression is altered during intervertebral disc degeneration and if hypoxia and HIF-1 regulate their expression in NP cells. AQP expression was measured in human tissues from different degenerative grades; regulation by hypoxia and HIF-1 was studied using promoter analysis and gain- and loss-of-function experiments. We show that both AQPs are expressed in the disc and that mRNA and protein levels decline with human disease severity. Bioinformatic analyses of AQP promoters showed multiple evolutionarily conserved HREs. Surprisingly, hypoxia failed to induce promoter activity or expression of either AQP. While genomic chromatin immunoprecipitation showed limited binding of HIF-1α to conserved HREs, their mutation did not suppress promoter activities. Stable HIF-1α suppression significantly decreased mRNA and protein levels of both AQPs, but HIF-1α failed to induce AQP levels following accumulation. Together, our results demonstrate that AQP1 and AQP5 expression is sensitive to human disc degeneration and that HIF-1α uniquely maintains basal expression of both AQPs in NP cells, independent of oxemic tension and HIF-1 binding to promoter HREs. Diminished HIF-1 activity during degeneration may suppress AQP levels in NP cells, compromising their ability to respond to extracellular osmolarity changes.

## INTRODUCTION

The intervertebral disc is a specialized organ that allows for normal biomechanical function of the spine. Each disc contains an outer fibrocartilagenous annulus fibrosus (AF), surrounding a gelatinous nucleus pulposus (NP). Two features uniquely characterizing the NP are hypoxia [1, 2] and elevated osmolarity yielding high tissue water content. Previous reports have demonstrated a constitutive expression of HIF-1α and HIF-2α in the NP [3-5], which control glycolytic metabolism, matrix production, and cell survival under hypoxic conditions [6-8]. High concentration of proteoglycans whose glycosaminoglycan side-chains are hydrophilic promotes water retention by the NP, allowing the disc to resist compressive loads and permit spinal motion [9-12]. NP cells experience daily fluctuations in water content, with measurements showing 8% water loss upon loading, highlighting the importance of controlling water transport to withstand osmotic and volumetric changes in these cells [12]. With aging, the intervertebral discs are highly susceptible to degeneration, characterized by loss of swelling pressure and hydration of the NP and pain [13, 14]. Therefore, tight regulation of water transport in NP cells is of utmost importance for disc function.

The Aquaporins (AQPs) are a thirteen-member family of water channels, driven by osmotic gradient [15]. Aquaporin 1 (AQP1), the first family member to be described, is found in many secretory and osmotically active tissues [16]. Expression of AQPs 1-3 has been shown in the intervertebral disc [17, 18]. While AQP2 expression is responsive to osmotic changes in NP cells [18], AQP1 showed only a small increase at 5% O_2_ [19]. In other tissues, expression of many members of the Aquaporin family including AQPs 1 and 5 have been linked to oxygen tension and ΗΙF-1α expression [20-28]. Additional studies have identified HIF-1α as a key regulator of AQP 1 and 5 induction in retinal vascular endothelial, Schwann and lung epithelial cells [29-31]. Interestingly, Kaweida *et al.* [32] reported a suppressive effect of HIF-1α on AQP5 expression in lungs of mice exposed to hypoxia and in lung epithelial MLE-12 cells, indicating that hypoxic regulation of AQPs may be cell-type specific.

The goal of this study was to investigate whether AQP expression is sensitive to intervertebral disc degeneration and if physiological hypoxia and HIF-1α play a role in their regulation in NP cells. We show that both AQPs have prominent membrane localization in disc tissues. Importantly, unique to NP cells, while expression is not hypoxia sensitive, it requires HIF-1 for maintaining basal levels. Noteworthy, under hypoxia, the ability of HIF-1α to bind conserved HREs in AQP promoters is not required for driving expression.

## RESULTS

### Aquaporin 1 and 5 Expression Levels Correlate with Degenerative Grade in Human Intervertebral Discs

**Figure 1:**
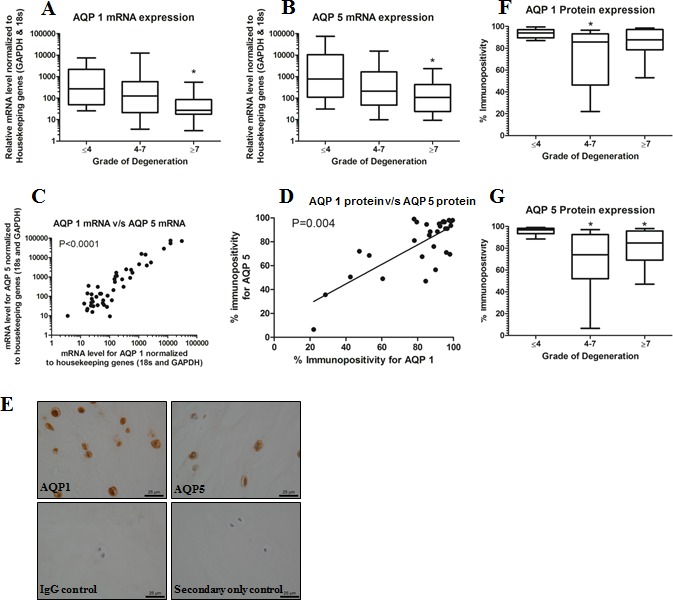
AQP expression decreases with degeneration in human intervertebral disc samples A and B, Levels of AQP1 (**A**) and AQP5 (**B**) mRNA decrease with disc degeneration (AQP 1:p=0.0018; AQP 5: p=0.0472). C and D, Linear correlation between mRNA (**C**) and protein (**D**) levels of AQPs 1 and 5 in disc samples, irrespective of histological grade (mRNA: p<0.0001; protein p=0.004). (**E**), Localization of AQP1 and AQP5 to cellular and nuclear membranes in NP cells. F and G, Percentage of cells immunopositive for AQP1 (**F**) or AQP5 (**G**) is decreased in degenerative discs. Significance in mid-grade degenerate discs (graded 4-7) compared to non-degenerative discs for AQP 1 (p=0.0484) and in both mid-grade and high-grade degenerative discs compared to non-degenerative discs for AQP5 (p = 0.003 and p=0.0397, respectively). In A, B, and C mRNA levels are multiplied by factor of 10^5^. Scale bar in E is 25 μm.

AQP1 and AQP5 mRNA levels decreased in degenerative NP compared to non-degenerative human NP tissues, difference reached significance in high grades of degeneration (graded ≥7) (AQP1: *p*=0.0018; AQP5: *p*=0.0472) (Fig. 1A and 1B). Interestingly, both mRNA levels and percentage of cells immunopostive for AQPs 1 and 5 in NP were correlated, demonstrating that discs with high levels of AQP1 also have high levels of AQP5 (mRNA: *p* < 0.0001; protein: *p*=0.04) (Fig. 1C and 1D). Moreover, AQP1 and AQP5 were localized to cell membranes of NP cells (Fig. 1E) in the majority of cells within non-degenerative discs (graded ≤4). Noteworthy, the percentage of immunopositive cells for both AQPs 1 and 5 decreased in degenerative discs, this was significant in mid-grade degenerative discs (graded 4-7) compared to non-degenerative discs for AQP1 (*p*=0.0484) (Fig. 1F) and in both mid-grade and high-grade degenerative discs compared to non-degenerative discs for AQP5 (*p* = 0.003 and *p* = 0.0397 respectively) (Fig. 1G).

### Aquaporins 1 and 5 are Expressed in the Normal Intervertebral Disc

**Figure 2:**
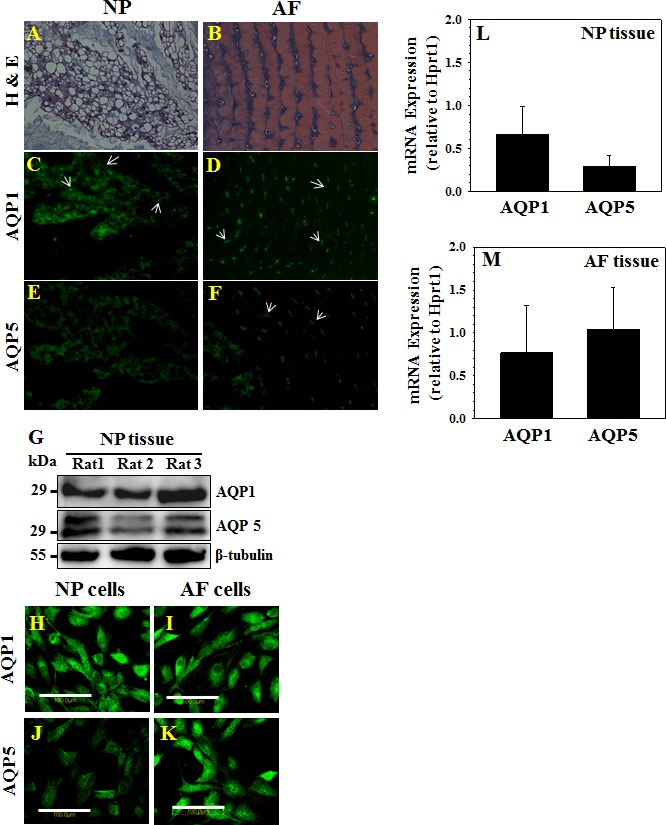
AQPs 1 and 5 are expressed in healthy rat disc Sections of rat intervertebral disc were stained with either H&E (**A, B**) or antibodies against AQP1 (**H, I**) or AQP5 (**E, F**). Note both AQPs are expressed in NP and AF tissues (arrows). In NP cells, prominent plasma membrane localization was seen. G, Western blot of AQPs from three rats demonstrates expression of 29 kDa AQP1 and AQP5 protein in NP tissues. Immunofluorescent detection of AQP1 and AQP5 protein in cultured NP and AF cells (H-K). L and M, Comparable levels of AQP 1 and 5 mRNA expression in rat NP (**L**) and AF (**M**) tissue samples was seen. Scale bar in H-K is 100 μm.

Since AQP expression was observed to be sensitive to disc degeneration, it was of interest to study their expression and regulation in native NP tissue. For this purpose, sections of NP and AF from rat intervertebral discs were first stained with antibody to detect either AQP1 (Fig. 2C and 2D) or AQP5 (Fig. 2E and 2F) localization. Additional sections were counterstained with H&E for assessment of general tissue morphology (Fig. 2A and 2B). Both AQP1 and AQP5 protein were detected in NP and AF tissues, with AQP1 showing more robust plasma membrane expression than AQP5 in NP sections. Protein expression of AQPs was further assessed in rat NP tissue and cultured NP and AF cells with Western blot analysis and immunofluorescence microscopy, respectively. As shown in Fig. 2G, both AQPs are expressed in freshly isolated NP tissue from three rats as evidenced by specific bands present at 29 kDa. Cultured NP and AF cells (Fig. 2H-2K) also expressed both AQPs. AQP mRNA expression was measured for both AQPs in NP (Fig. 2L) and AF (Fig. 2M) tissue isolated from three rats. All experimental data demonstrate a trend of similar expression of both AQPs 1 and 5 in NP cells and tissue.

### The Proximal Promoter Regions of AQP1 and AQP5 Contain Conserved Hypoxia Response Elements

**Figure 3:**
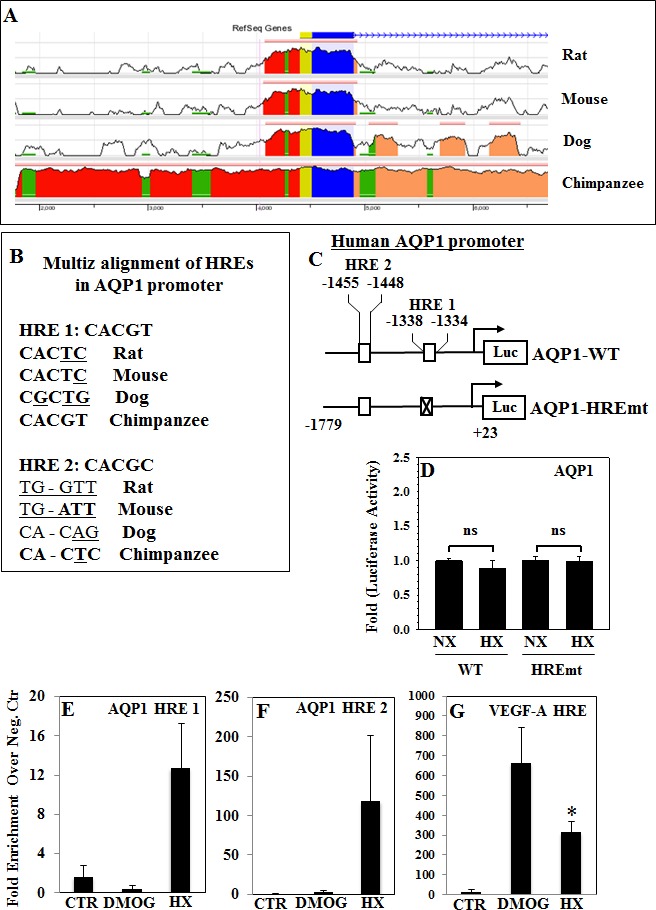
Functionality of conserved HREs in the promoter regions of the *AQP1* gene **A**, Analysis using the Evolutionary Conserved Regions Browser (http://ecrbrowser.dcode.org/) demonstrates sequence conservation between vertebrates in the 2 kb *AQP1* promoter. ECR threshold is set at 80%. *AQP1* promoter: *red*, 5′ UTR; *yellow*, exons; *blue*, introns; *peach*, transposons; and simple repeats, *green.* **B**, Multiz alignment of two HREs in the *AQP1* promoter to evaluate inter-species sequence homology. **C**, Schematic representation of wild-type (WT) and HRE-mutant (HREmt) *AQP1* promoter constructs. **D**, Promoter activity remains constant between normoxia (NX, 21% O_2_) and hypoxia (HX, 1% O_2_) in rat NP cells. ChIP results for HIF-1α binding to HREs in human NP cells untreated (CTR), treated with 2mM DMOG, or cultured under hypoxia for 24 h (HX). qRT-PCR was performed on ChIP samples using specific primers for AQP1 HRE 1 (**E**), AQP1 HRE 2 (**F**), and VEGF-A HRE (**G**). Data is represented as fold enrichment in HIF-1α binding over negative control primers, positive binding is defined as enrichment ≥ 5 fold.

**Figure 4:**
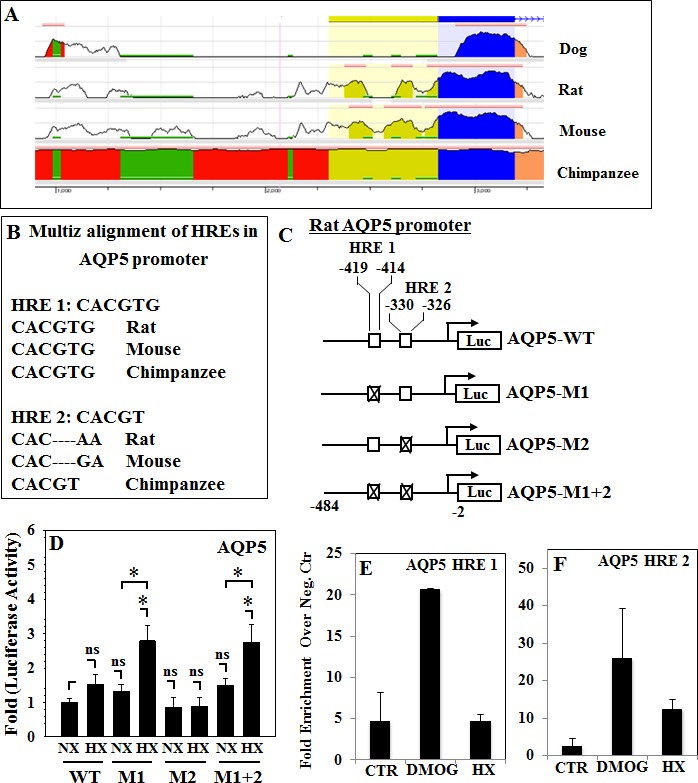
Functionality of conserved HREs in the promoter regions of the *AQP5* gene **A**, Analysis using the Evolutionary Conserved Regions Browser demonstrates sequence conservation between multiple species in the 1 kb *AQP5* promoter. **B**, Multiz alignment of two HREs in the *AQP5* promoter to evaluate inter-species sequence homology. **C**, Schematic representation of wild-type (AQP5-WT) and HRE-mutant (AQP5-M1, -M2, M1+2) promoter constructs. **D**, Similar promoter activity was observed in rat NP cells cultured under normoxia (NX, 21% O_2_) or hypoxia (HX, 1% O_2_). ChIP results for HIF-1α binding to HREs in human NP cells untreated (CTR), treated with 2 mM DMOG, or cultured under hypoxia for 24 h (HX). qRT-PCR was performed on samples using specific primers for AQP5 HRE 1 (**E**) and AQP5 HRE 2 (**F**). Data is represented as fold enrichment in HIF-1α binding over negative control primers positive binding is defined as enrichment ≥ 5 fold.

To define the regulatory mechanism controlling AQP expression in NP cells in hypoxia, the promoter regions of *AQP1* and *AQP5* were analyzed. First, the ECR Browser (http://ecrbrowser.dcode.org/) was used to evaluate the level of interspecies sequence conservation across the entire *AQP1* gene (Fig. 3A), revealing high conservation of exonic sequences (blue). Next, 1.5 kb of the human *AQP1* promoter was scanned for the presence of hypoxia responsive elements (HREs) using the JASPAR core database (http://jaspar.genereg.net/). Two putative HREs: HRE 1 at −1338/−1334 bp and HRE 2 at −1455/−1448 bp of the human promoter, were identified (Fig. 3B). Multiz alignment was also performed for both HREs. As shown in Fig. 3B, HRE 1 demonstrates high level of sequence conservation between multiples vertebrates. Similarly, evaluation of AQP5 gene sequence homology using the ECR Browser also showed high conservation of exonic regions (blue) and UTRs (yellow) (Fig. 4A). Two HREs sites were returned by the JASPAR database, in the rat *AQP5* promoter they are located at HRE 1 at −419/−414 bp and HRE 2 at −330/−326 bp (Fig. 4B). Again, Multiz alignment of HREs of *AQP5* promoter showed high sequence conservation between shown species (Fig. 4B).

### Promoter Activities of AQP1 and AQP5 are Not Hypoxia-Inducible in NP Cells

To determine regulation of promoter activity by hypoxia, rat NP cells were transfected with reporters containing either AQP1 or AQP5 promoters and luciferase activity was measured under normoxia and hypoxia (1% O_2_). Hypoxic culture failed to induce activity of either the wild-type AQP1 (Fig. 3D) or the wild-type AQP5 (Fig. 4D) promoters.

### Transcription AQP1 and AQP5 is Independent of HIF-1α Binding to HREs in Promoters

The hypothesis that HIF-1 regulates AQP transcription via HRE sites was specifically tested first by measuring activity of constructs harboring mutations of the highly conserved HRE sites (HRE1 in *AQP1* and HREs 1 and 2 in the *AQP5* promoter) in rat NP cells. As shown in Fig. 3D, mutation of HRE 1 in the *AQP1* promoter did not alter its activity in response to hypoxia. Similarly, mutation of HRE 2 in the *AQP5* promoter did not affect response to hypoxia. Mutation of HRE 1 in the *AQP5* promoter, either alone or in concert with HRE 2 mutation, resulted in an increase in promoter activity under hypoxia. To investigate whether these conserved HREs bind HIF-1α and whether binding was dependent upon oxygen tension, genomic chromatin immunoprecipitation for HIF-1α was performed in human NP cells cultured in normoxia with or without DMOG, a prolyl hydroxylase inhibitor or under hypoxia. Primers were designed to specifically amplify only the region containing a single AQP HRE, previously characterized VEGF-A HRE was used as positive control [36]. HIF-1α bound to a limited extent only to HRE 1 and HRE 2 of *AQP1* promoter under hypoxic conditions, no enrichment in binding over negative control primers was seen under normoxia with or without DMOG treatment (Fig. 3E and 3F). On the other hand, enrichment in HIF-1α binding to both HREs in *AQP5* promoter with DMOG treatment and to HRE 2 under hypoxia was seen (Fig. 4E and 4F). Despite the fact that HIF-1α could interact with all four HREs to varying degree under at least one experimental condition, levels of binding were several-fold lower than that of HIF-1α with the known VEGF-A HRE (Fig. 3G).

### Hypoxia Fails to Induce Expression of AQP 1 and AQP5 in NP Cells

**Figure 5:**
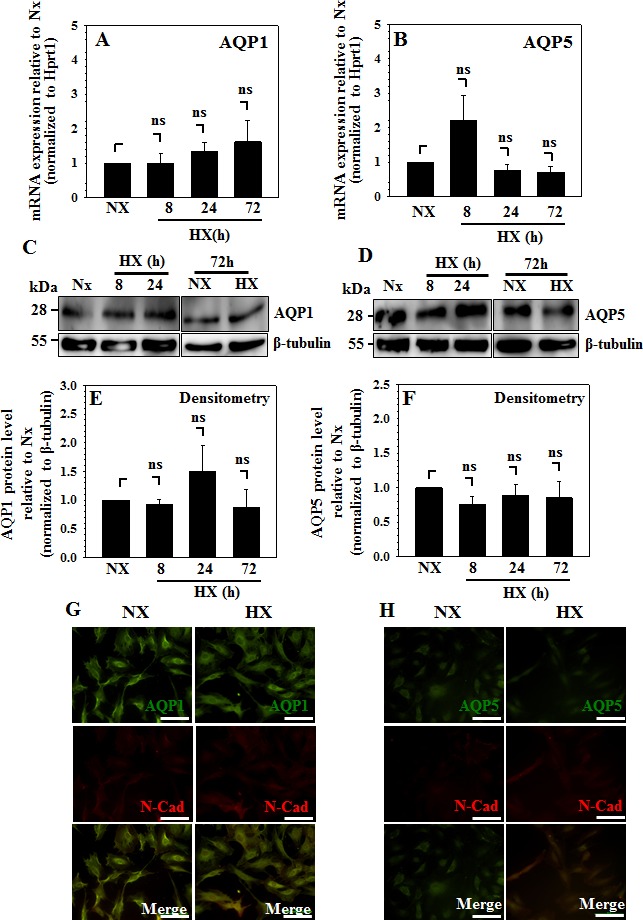
Expression of AQPs 1 and 5 is refractory to hypoxia in NP cells A and B, AQP1 (**A**) and AQP5 (**B**) mRNA levels in rat NP cells cultured in normoxia (NX, 21% O_2_) or hypoxia (HX, 1% O_2_) for 8-72 h. mRNA expression showed no significant change under hypoxia. C-F, Western blot and corresponding Densitometric analysis shows that protein expression of AQP1 (**C**, **E**) and AQP5 (**D**, **F**) in rat NP cells is refractory to hypoxic conditions. G and H, Immunofluorescent detection of AQP1 and AQP5 in NP cells following 24 h in normoxia (NX) or hypoxia (HX). N-Cadherin is used as a marker for plasma membrane staining. Scale bar in G and H is 100 μm.

To test the effect of hypoxia on expression levels of AQP1 and AQP5, rat NP cells were cultured in 1% O_2_ for 8 to 72 h and mRNA and protein levels were measured. As shown in Fig. 5A and 5B, hypoxic culture did not significantly affect mRNA expression of either AQP in NP cells. Western blots and corresponding densitometric analyses confirm that hypoxia does not affect protein expression of either AQP1 (Fig. 5C and 5E) or AQP5 (Fig. 5D and 5F). To investigate possible changes in protein localization in response to hypoxia, cells were incubated in hypoxia for 24 h before immunofluorescent staining with antibodies directed against either AQP1 or AQP5 along with N-Cadherin. Fig. 5G-5H shows that expression and localization of both AQPs remain unaffected by hypoxia.

### HIF-1α is Necessary for Basal Expression of Aquaporins 1 and 5 in NP Cells

**Figure 6:**
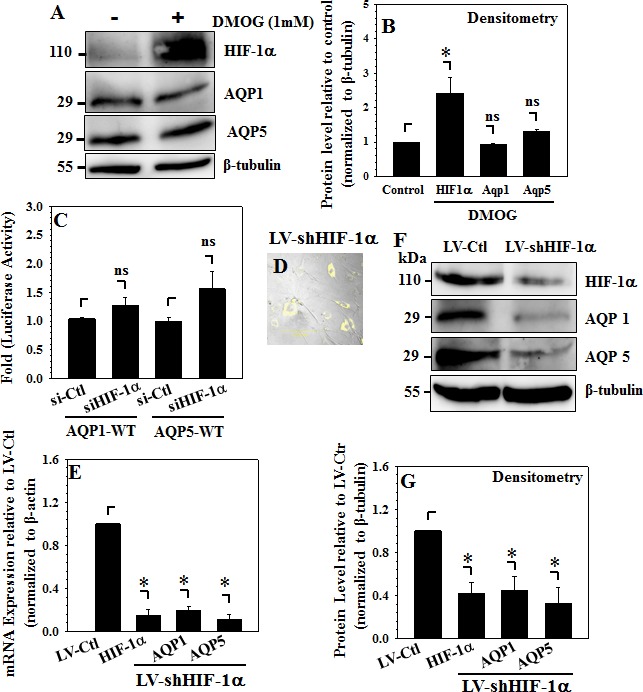
HIF-1α maintains basal AQP expression in NP cells A and B, Western blot (**A**) and corresponding densitometric analysis (**B**) show that treatment of rat NP cells with DMOG (1mM) has no effect on levels of AQP1 and AQP5, as expected accumulation of HIF-1α protein by DMOG is evident. **(C)**, Suppression of HIF-1α expression by co-transfection with Si-HIF-1α does not significantly change activities of AQP1 and AQP5 promoters in comparison to rat NP cell transfected with control siRNA. **(D)**, YFP expression demonstrates efficient transduction of human NP cells infected with lentivirus encoding both HIF-1α shRNA and YFP. **(E)**, Stable Silencing of HIF-1α by lentiviral Sh-HIF-1α causes strong decrease in basal levels of both AQP 1 and AQP5 mRNA in human NP cells. As expected, HIF-1α mRNA levels were significantly lower in silenced cells compared to cells that received control shRNA. F and G, Western blot (**F**) and corresponding densitometric analysis (**G**) shows that HIF-1α silencing in human NP cells results in robust decrease in both AQP 1 and AQP5 protein levels.

The effect of HIF-1 on AQP expression in rat NP cells was further investigated using gain- and loss-of-function studies. To block prolyl hydroxylase activity and accumulate HIF-1α protein, cells were treated with 1 mM DMOG. Western blot and corresponding densitometric analysis (Fig. 6A and 6B) show that, while DMOG treatment led to accumulation of HIF-1α protein, expression levels of AQP1 and AQP5 remained unchanged. Next, rat NP cells were co-transfected with the wild-type reporter constructs of either the AQP1 or the AQP5 promoter along with siHIF-1α or a control siRNA. Results clearly showed that suppression of HIF-1 by siHIF-1α did not affect promoter activity of either wild-type AQP promoter construct (Fig. 6C). To assess effect of stable HIF-1 suppression on AQP expression, human NP cells were transduced by lentivirus co-expressing shHIF-1α and YFP. As shown in Fig. 6D, YFP signal indicated that lentiviral delivery resulted in high infection rates and a robust transgene expression in NP cells. As expected, shHIF-1α significantly decreased mRNA expression of HIF-1α compared to sh-control (Fig. 6E-6G). Importantly, silencing of HIF-1α resulted in dramatic decrease in mRNA expression of both AQP1 and AQP5 in human NP cells (Fig. 6E). Moreover, HIF-1α knockdown resulted in significant reduction in protein levels of both AQP1 and AQP5 (Fig. 6G).

## DISCUSSION

This study demonstrates the importance of maintenance of AQP levels for human disc health and defines the unique HIF-1α-dependent regulation of AQPs 1 and 5 in NP cells. We show for the first time that AQP5 is expressed in the disc and that expression levels of both of these AQPs are correlated with severity of human disc disease. Unlike reports in other cell types, we show that expression of these AQPs is not hypoxia-inducible in NP cells. Importantly, our results show that, while HIF-1α positively regulates basal expression of both AQPs, it does so independently of conserved HREs identified in their promoters. Restoration of AQP levels and osmoregulatory capacity of cells by modulating HIF-1 signaling may represent a novel therapeutic strategy to mitigate effects of disc disease.

Biomechanical function of the intervertebral disc is intricately linked to NP osmotic properties and the ability of matrix to bind water. Since AQPs are crucial in maintaining cellular water homeostasis under dynamic osmotic conditions and have been shown to co-transport many important metabolites such as CO_2_ and O_2_ [37, 38], it was important to investigate if their expression is sensitive to disc degeneration. Our results clearly show that levels of both AQPs are lower in NP tissues with progressive degeneration. These results provide further support to observations by Wang *et al.* [19], who showed differential expression of AQP1 in young versus aged rabbit discs, suggesting a possible role in age-related degeneration of the disc. Importantly, AQP 1 and 5 expression levels are sensitive to changes in extracellular osmolarity [31, 39, 40]. Likewise, AQP2 expression in NP cells is osmo-dependent [18]. Thus, decreased levels of these and other AQPs possibly indicate loss of osmoregulatory capacity of NP cells and may result in compromised cell function. Relevant to this discussion, clinical studies of low back pain patients have shown a strong inverse relationship between pain and T1ρ MRI and opening pressure, measures of disc hydration and osmotic pressure, respectively [41]. These changes in tissue osmotic properties are largely attributed to loss of aggrecan and other GAGs as catabolic activities overwhelm normal anabolism of the matrix [42, 43]. It is thus possible that decreased AQPs may be a reflection of changing osmotic status of the tissue. Nevertheless, our results point to AQP loss as another critical step that characterizes this pathogenic process.

Interestingly, our results show a strong correlation between expression of AQP1 and AQP5 in NP, independent of degenerative grade, suggesting a common regulatory mechanism. It was therefore of high interest to investigate how expression of AQPs 1 and 5 is regulated in healthy tissues. Our results show robust expression of not only AQP1 but also AQP5 in discal tissues and cultured cells. This finding was in line with previous reports showing presence of AQPs 1-3 in the disc [17, 18], while AQPs 1 and 5 are expressed in cartilage, a tissue that also experiences low-oxygen, high-osmolarity environment similar to that of the NP [44-46]. Expression of multiple AQPs in NP is not surprising, as water balance in this osmotically challenging tissue must be tightly controlled for cell survival and functional activities.

Physiological hypoxia, a distinguishing feature of the NP environment, is a critical regulator of cell survival and function of these cells [3, 6-8, 47]. We therefore asked the question of whether hypoxia controls the expression of AQPs 1 and 5 in NP cells. Previous studies have shown that expression of AQP1 and 5 is sensitive to hypoxia and that activity of their proximal promoters is controlled by HIF-1α [29, 31]. In contrast to these reports, our results show that promoter activity is insensitive to hypoxia. Moreover, while promoters contain multiple HREs, mutation of highly conserved HRE 1 (−1338/−1334 bp) of AQP1 and HRE 2 (−330/−326 bp) of AQP5 had no impact on their activities suggesting that these motifs are not required for regulation. Our lab has recently reported unique regulation of CCN2, HSP70, and GlcAT-1 [6, 47, 48] by HIF-1α in NP cells. In the case of CCN2, deletion and mutation analyses of HREs indicated indirect regulation by HIF-1α [47], similar to what we have presented here for AQP1 and AQP5. These data support the notion that, in the unique NP environment, HIF acts as a regulator either in a complex that does not require HIF-DNA binding or at a level other than transcription. In stark contrast to NP cells, HRE 1 of AQP1 has been shown to modulate HIF-1α response in human retinal vascular endothelial cells (HRVECs) [29], while HREs in AQP5 promoter control HIF-1α dependent activation in mouse lung epithelial cell line MLE-15 [31]. These results raised an important question: Can AQP-HREs even interact and bind to HIF-1α in NP cells? Results of genomic ChIP analysis clearly showed that, while a low level binding could be detected either in presence of DMOG or under hypoxia, it is much lower than binding to the validated HRE in promoter of VEGF-A, an hypoxia and HIF-inducible gene in NP cells [5, 36]. Interestingly, a distinct pattern of HIF-1-HRE interaction was observed for individual AQPs implying that for AQP1 hypoxia-specific co-factors seemed necessary for HRE binding as no enrichment was observed with DMOG, while high levels of HIF-1α from DMOG are sufficient to force binding to the AQP5 HREs. Supporting our mutagenesis and ChIP analysis, transfection results using SiHIF-1α with AQP reporters indicated that depletion of HIF-1α from the system had no effect on promoter activities.

While results of promoter experiments suggested that hypoxia and HIF-1 do not contribute to controlling activities of reporters, it was likely that the regulatory motif(s) could lie outside of these promoter fragments or the regulation was at post-transcriptional or translational level. To investigate this possibility hypoxic expression of AQPs was assessed. Again, highlighting unique response of NP cells, we observed lack of hypoxic induction of either AQPs at the mRNA or protein level. Since in NP cells HIF-1α levels are refractory to oxygen-mediated degradation, there is no appreciable increase in HIF-1α level in hypoxia. Thus, to investigate whether elevation in HIF-1α levels can induce AQP expression, we treated cells with DMOG to force accumulation. Notably, despite dramatic increase in HIF-1α protein levels, AQP levels remained unaltered. This is in agreement with our results that showed lack of AQP inducibility by increased HIF-1 activity under hypoxia. Finally, we determined whether HIF-1α was necessary for maintenance of basal expression of AQPs in NP cells. Results of stable suppression of HIF-1α showed approximately 80% and 60% decrease in both AQPs' mRNA and protein levels, respectively. These results clearly suggest that AQP1 and 5 are HIF-1 target genes. It is also possible that the expression of AQPs is indirectly controlled through other HIF-1 targets. That hypoxia or DMOG does not induce AQP expression in NP cells may be partially attributed to the fact that sufficient HIF-1α activity is always present to maintain optimal levels of AQPs in NP cells.

There is indirect evidence to suggest that changes in local oxygen tension resulting from annular fissures during degeneration may compromise HIF-1α activity in the NP possibly due to dysregulation of protein turnover and co-factor expression [4, 7, 49, 50]. Notably, expression of PHD3 that serves as important HIF-1 co-factor decreases with degeneration, implying diminished HIF-1α activity [35]. Based on these previous reports and our current findings, it is plausible that changes in HIF-1α expression and/or activity with degeneration may result in decreased AQP expression, causing further exacerbation of disease. The potential role of AQPs in the exacerbation of intervertebral disc degeneration suggests therapies targeted at restoring AQP expression and function within degenerative discs may provide a new strategy for reestablishing the osmotic balance within the IVD.

## MATERIALS AND METHODS

### Human tissue collection and grading for investigation of AQP expression

Human lumbar IVD tissues were obtained at surgery or post-mortem (PM) examination with informed consent of the patient or relatives (Sheffield Research Ethics Committee #09/H1308/70). Seven PM IVDs were recovered from two donors, comprising intact IVDs within the complete motion segment from which the IVDs were removed. 73 surgical IVD tissue samples were obtained from 73 patients undergoing micro-discectomy procedures for treatment of low back/neck pain and root pain as caused by prolapse of the IVD (Supplemental Table 1 for sample details). NP tissue was very carefully separated from the AF and endplate fragments under stereomicroscope [33] and was divided into two - half was fixed in 10% neutral buffered formalin and processed for histological and immunohistochemical examination. The remaining NP tissue was used for RNA isolation. H&E stained sections, used to score degree of morphological degeneration, were scored numerically 0-3 for four features as reported previously [33]. Gene expression samples were classified as non-degenerative (≤ 3.9), mid-grade degenerative (4-6.9) and high grade degenerative (≥7) based on histological examination. AF tissues were not included in any of the analyses due to chances of cross contamination from extraneous connective tissues, endplate fragments and blood. Grading was performed independently by two researchers and grades were averaged.

### Isolation of NP and AF cells and treatments of cells

Rat NP and AF and human NP (2 samples, 33 yrs. (F) Autopsy, grade 1 and 39 yrs. (M), Surgical waste, grade 2) cells were isolated using a method previously described by Risbud *et al.* [4] Collection of animal and human tissues for cell isolation was performed as per approved protocols by Jefferson's IACUC and IRB respectively. Cells were passaged and maintained in monolayer in Dulbecco's Modified Eagles Medium (DMEM) together with 10% fetal bovine serum (FBS) supplemented with antibiotics. For hypoxic culture NP cells were maintained in a Hypoxia Work Station (Invivo2 300, Ruskin, UK) with a mixture of 1% O_2_, 5% CO_2_, and 94% N_2_ for 8 to 72 h. In some experiments, cells were treated with 1-2mM dimethyl oxalylglycine (DMOG).

### Immunohistochemical analysis

Human discs: Immunohistochemistry confirmed and localized production of AQPs 1 and 5 in 29 IVDs; 7 PM and 22 surgical samples (Supplementary Table 1). Tissue sections were dewaxed, rehydrated, endogenous peroxidases quenched. Samples were blocked in rabbit (AQP1) or goat (AQP5) serum, then incubated overnight at 4°C with mouse anti-human AQP1 (1:10), or rabbit anti-human AQP5 (1:10), with pre-immune mouse and rabbit IgG (Abcam) used as negative control at equal IgG concentrations. After washing, AQP1 sections were incubated with biotinylated rabbit anti-mouse antiserum (1:400; Abcam) and AQP5 sections incubated with biotinylated goat anti-rabbit antiserum (1:500; Abcam) and binding detected by formation of streptavadin-biotin complex (Vector Laboratories, Peterborough, UK) with 3,3′-diaminobenzidine tetrahydrochloride solution (Sigma-Aldrich). Sections were counterstained with Mayers Haematoxylin (Leica Microsystems), dehydrated, cleared and mounted in Pertex (Leica). Sections were visualized and images captured using an Olympus BX60 microscope and QCapture Pro v8.0 software (MediaCybernetics, Marlow, UK). Random fields of view were assessed for immunopositive cells until a total of 200 NP cells were counted, the number of immunopositive cells was expressed as a percentage of the total count.

Rat discs: Transverse and coronal sections (6-8 μm) of rat spines were deparaffinized in xylene, rehydrated through graded ethanol and stained with Alcian Blue, eosin and hematoxylin. Sections were blocked with goat serum (Invitrogen) and incubated with the AQP1 (1:100; Millipore) or AQP5 (1:100; Calbiochem) antibodies in 2% bovine serum albumin at 4 °C overnight. After washing, the bound primary antibody was incubated with Alexa fluor-488 conjugated secondary antibody (1:50; Invitrogen) and imaged using a fluorescence microscope (Nikon, Japan).

### Plasmids and reagents

Human AQP1 (AQP1-WT, AQP1-HREmt) and AQP5 (AQP5-WT, AQP5-M1, AQP5-M2, AQP5-M1+2) constructs were gift from Drs. K. Sakurai, Chiba University, Japan [29] and Z. Borok, University of Southern California [31] respectively. SiHIF-1α plasmid (#21103), from Dr. C. Cepko was from Addgene. For internal transfection control, vector pRL-TK (Promega) containing *Renilla reniformis* luciferase gene was used.

### Real-time RT-PCR

Total DNA-free RNA was extracted from NP cells using RNAeasy mini columns (Qiagen), and cDNA made using EcoDry premix (Clontech). cDNA and gene-specific primers (IDT, IA) were added to SYBR Green master mixture and mRNA expression quantified using Step-One Plus Real-time PCR System (Applied Biosystems). Hprt1 was used to normalize gene expression. For human NP tissues, extracted tissue RNA was DNase treated (Qiagen) and purified using Qiagen MinElute Cleanup kit before cDNA synthesis using MMLV Reverse Transcriptase (Bioline, London, UK) and random hexamers. Real-time PCR analysis was performed using pre-designed, FAM-labeled Taqman^®^ Gene Expression Assays, GAPDH and 18s were used to normalize mRNA (Applied Biosystems). 58 IVDs were used for this component of the study (Supplementary Table 1).

### Chromatin immunoprecipitation

Human NP cells in 10-cm plates were treated with or without 2 mM DMOG for 24 h in normoxia or placed in hypoxia for 24 h. ChIP assay was performed using ChIP-IT® High Sensitivity kit (Active Motif, CA) following manufacturers recommendations and as reported previously [34]. DNA complexes were immunoprecipitated using HIF-1α antibody (Abcam) overnight at 4°C followed by binding to protein G-agarose beads. Cross-links were reversed by treatment with proteinase K and heat, and DNA was purified using DNA purification elution buffer. Real-time PCR analysis was performed using ChIP-IT® qPCR analysis kit (Active Motif). Negative control and standard curve primers used were provided with kit. Ct values for samples were recorded and the data were normalized based on primer efficiency, input DNA Ct values, amount of chromatin, and resuspension volume, based on manufacturer's recommendations.

### Western blotting

Cells were placed on ice following treatment and washed with ice-cold HBSS. All buffers included 1X protease inhibitor cocktail (Roche), NaF (5 mM) and Na3VO4 (200 μM). Total cell proteins were resolved on 8-12% SDS-polyacrylamide gels and transferred by electroblotting to PVDF membranes (Bio-Rad, CA). Membranes were blocked with 5% non-fat dry milk in TBST (50 mM Tris, pH 7.6, 150 mM NaCl, 0.1% tween 20) and incubated overnight at 4°C in blocking buffer with the AQP1 (1:1500) (Millipore), AQP5 (1:1000) (Millipore), or anti-β-tubulin antibody (1:2000, DSHB). Immunolabeling was detected with ECL reagent (Amersham). Blot intensity was determined using densitometric analysis (ImageQuant).

### Immunofluorescence microscopy

Cells were plated in 96-well plates (4 × 10^3^/well) then cultured in normoxia or hypoxia. After incubation, cells were fixed with 4% paraformaldehyde, permeabilized with 0.2% triton-X 100 in PBS for 10 min, blocked with PBS containing 5% FBS, and incubated with antibodies against AQP1, AQP5 (1:100, Millipore), or N-Cadherin (1:800 BD Transduction Laboratories) at 4°C overnight. For negative control, isotype IgG under similar conditions was used. After washing, the cells were incubated with Alexa fluor-488 conjugated anti-rabbit or Alexa-fluor-594 conjugated anti-mouse secondary antibody (1:50, Invitrogen) and following washing imaged using a fluorescence microscope (Nikon, Japan).

### Transfections and dual luciferase assay

Cells were transferred to 48-well plates (2 × 10^4^ cells/well) one day pre-transfection. To measure hypoxic effect, cells were transfected with 250 ng of AQP reporters with 250 ng pRL-TK plasmid and cultured in normoxia or hypoxia for 24 h. For silencing, siHIF-1α (200 ng) or control siRNA (200 ng) was co-transfected with AQP reporter and pRLTK. In all experiments plasmids were premixed with the transfection reagent, LipofectAMINE 2000 (Invitrogen). The next day, cells were harvested and Dual-Luciferase™ reporter assay system (Promega) was used for measurements of firefly and Renilla luciferase activities using a luminometer (TD-20/20, Turner Designs, CA).

### Lentiviral production and transduction

HEK 293T cells seeded in 10-cm plates (1.3 × 10^6^ cells/plate) were transfected with 9 μg of pLKO.1-Sh-Ctr (LV-Ctl) or LV-shHIF-1α plasmids along with 6 μg of psPAX2 and 3 μg of pMD2.G [35]. After 16 h, transfection media was removed and replaced with DMEM with 5% heat-inactivated FBS. Lentiviral particles were harvested at 48 and 60 h post-transfection. NP cells in 10-cm plates were transduced with 8 ml of conditioned media containing viral particles and 6 μg/ml of polybrene. After 24 h, conditioned media was removed and replaced with DMEM, 5% FBS and cells harvested for analysis 5 days after transduction.

### Statistical analysis

All measurements were performed in triplicate. Results are presented as the mean ± S.E. Differences between groups were assessed by ANOVA and Student's T-test. *P*-values < 0.05 were considered statistically significant. Human expression data was non-parametric, thus Kruskal-Wallis test with post-hoc analysis by Conover-Inman test was used to determine significance between 2^ΔCT^ values and percentage immunopositivity between groups. Spearman's rank correlation was used to determine correlations between gene and protein expression for different targets.

## SUPPLEMENTARY MATERIAL AND TABLE


