# Supplementary material for: Aquaporin 1 and 5 expression decreases during human intervertebral disc degeneration: novel HIF-1-mediated regulation of aquaporins in NP cells
Source: Oncotarget. 2015 Mar 20;6(14):11945–58. doi: 10.18632/oncotarget.3631 (PMC4494915; doi:10.18632/oncotarget.3631)
Supplement: Supplementary file 1 [file oncotarget-06-11945-s001.pdf]

Aquaporin 1 and 5 expression decreases during human intervertebral disc degeneration: Novel HIF-1-mediated regulation of aquaporins in NP cells

Supplementary Material

Supplemental Table 1: Details for human tissues used in Real-time PCR and Immunohistochemical analysis. PM: Post mortem samples; M: Male; F: Female; L: Lumbar; C: Cervical.

	Source	Sex	Age	IVD Level	Histological Grade	qPCR	IHC
1	Surgical	M	49	L2/3	1	✓	
2	Surgical	M	32	L5/S1	2	✓	
3	PM	M	45	L3/4	2	✓	✓
4	Surgical	M	51	L4/5	2	✓	
5	Surgical	M	42	L5/S1	2.5		✓
6	Surgical	M	23	L4/5	3	✓	
7	Surgical	M	35	L5/S1	3	✓	
8	Surgical	M	20	L4/5	3	✓	
9	PM	M	45	L4/5	3	✓	✓
10	Surgical	M	42	L5/S1	3	✓	
11	Surgical	F	39	L5/S1	3		✓
12	Surgical	F	46	L4/5	3	✓	
13	Surgical	M	32	L5/S1	3	✓	✓
14	Surgical	F	25	L4/5	3	✓	
15	Surgical	F	40	L4/5	3	✓	
16	Surgical	M	39	L5/S1	3.35	✓	
17	Surgical	M	43	L5/S1	3.5		✓
18	Surgical	M	42	L4/5	4	✓	
19	Surgical	M	25	L4/5	4	✓	
20	Surgical	M	45	L5/S1	4	✓	
21	Surgical	F	26	L4/5	4	✓	
22	Surgical	F	44	L5/S1	4	✓	✓
23	Surgical	F	32	L5/S1	4	✓	
24	Surgical	M	45	C5/C6	4.5		✓
25	Surgical	F	26	L5/S1	4.65		✓
26	Surgical	F	39	L5/S1	5	✓	
27	Surgical	F	52	L4/5	5		✓
28	Surgical	F	28	L4/5	5		✓
29	Surgical	M	36	L5/S1	5		✓
30	Surgical	M	47	L5/S1	5		✓
31	Surgical	F	36	L5/S1	5	✓	
32	PM	M	45	L5/S1	5.1		✓
33	Surgical	M	-	L4/5	5.1		✓
34	Surgical	M	49	L2/3	5.1		✓
35	Surgical	M	39	L5/S1	5.15	✓	
36	Surgical	F	48	L5/S1	5.3	✓	
37	Surgical	F	48	L5/S1	5.85	✓	
38	Surgical	M	63	L3/4	6	✓	✓
39	Surgical	M	27	C3/4	6	✓	
40	Surgical	M	63	C5/6	6.1	✓	
41	Surgical	M	31	L5/S1	6.15	✓	

42	Surgical	M	47	L5/S1	6.16	✓	✓
43	Surgical	F	33	L5/S1	6.5		✓
44	Surgical	M	36	L4/5	6.5		✓
45	Surgical	F	65	L3/4	6.5	✓	
46	Surgical	F	63	L4/5	6.7	✓	
47	PM	F	74	L1/2	6.8		✓
48	Surgical	M	34	L4/5	6.8		✓
49	Surgical	M	77	L5/S1	6.85	✓	
50	Surgical	F	40	L5/S1	7	✓	
51	Surgical	F	34	L4/5	7		✓
52	PM	F	74	L4/5	7		✓
53	Surgical	F	38	L5/S1	7	✓	
54	Surgical	F	44	L5/S1	7	✓	
55	Surgical	F	43	L5/S1	7	✓	
56	Surgical	M	41	L3/4	7	✓	
57	Surgical	M	39	L5/S1	7	✓	
58	Surgical	F	38	L4/5	7	✓	
59	Surgical	F	41	L5/S1	7.5		✓
60	Surgical	M	-	L4/5	7.7		✓
61	Surgical	F	29	L4/5	8	✓	
62	Surgical	F	36	L5/S1	8	✓	
63	Surgical	F	28	L5/S1	8	✓	
64	Surgical	M	36	L5/S1	8	✓	
65	Surgical	M	33	L4/5	8	✓	
66	Surgical	F	-	L5/S1	8	✓	
67	Surgical	F	38	L5/S1	8	✓	
68	Surgical	F	50	L4/5	8	✓	
69	Surgical	F	29	L5/S1	8	✓	
70	Surgical	M	41	L4/5	8	✓	
71	Surgical	M	39	L4/5	8.15	✓	
72	PM	F	74	L3/4	8.8		✓
73	Surgical	F	33	L5/S1	9	✓	
74	Surgical	F	28	L4/5	9	✓	
75	Surgical	F	43	L5/S1	9	✓	✓
76	Surgical	F	38	C6/7	9	✓	
77	PM	F	74	L5/S1	9.1		✓
78	Surgical	M	48	L4/5	10	✓	
79	Surgical	F	26	L5/S1	11	✓	
80	Surgical	F	35	L4/5	11	✓	
